# Wheat blast: from its origins in South America to its emergence as a global threat

**DOI:** 10.1111/mpp.12747

**Published:** 2018-10-24

**Authors:** Paulo Cezar Ceresini, Vanina Lilián Castroagudín, Fabrício Ávila Rodrigues, Jonas Alberto Rios, Carlos Eduardo Aucique‐Pérez, Silvino Intra Moreira, Daniel Croll, Eduardo Alves, Giselle de Carvalho, João Leodato Nunes Maciel, Bruce Alan McDonald

**Affiliations:** ^1^ Department of Crop Protection, Agricultural Engineering, and Soils UNESP University of São Paulo State Ilha Solteira Campus São Paulo Brazil 15385-000; ^2^ Department of Plant Pathology, Lab. of Host‐Parasite Interaction UFV Federal University of Viçosa Viçosa Minas Gerais Brazil 36570-000; ^3^ Department of Plant Pathology UFLA Federal University of Lavras Lavras Minas Gerais Brazil 37200-000; ^4^ Laboratory of Evolutionary Genetics, Institute of Biology University of Neuchâtel Neuchâtel Switzerland CH-2000; ^5^ Brazilian Agriculture Research Corporation, Embrapa Wheat (Embrapa Trigo) Passo Fundo Rio Grande do Sul Brazil 99050-970; ^6^ Plant Pathology Group, Institute of Integrative Biology Swiss Federal Institute of Technology, ETH Zurich Zurich Switzerland CH-8092; ^7^ Present address: Department of Plant Pathology University of Arkansas AR USA

**Keywords:** infection physiology, integrated disease management, international quarantine, origin and diversification, population biology and epidemiology, *Pyricularia graminis‐tritici*

## Abstract

Wheat blast was first reported in Brazil in 1985. It spread rapidly across the wheat cropping areas of Brazil to become the most important biotic constraint on wheat production in the region. The alarming appearance of wheat blast in Bangladesh in 2016 greatly increased the urgency to understand this disease, including its causes and consequences. Here, we summarize the current state of knowledge of wheat blast and aim to identify the most important gaps in our understanding of the disease. We also propose a research agenda that aims to improve the management of wheat blast and limit its threat to global wheat production.

## The Emergence and Spread of Wheat Blast

**Figure 1:**
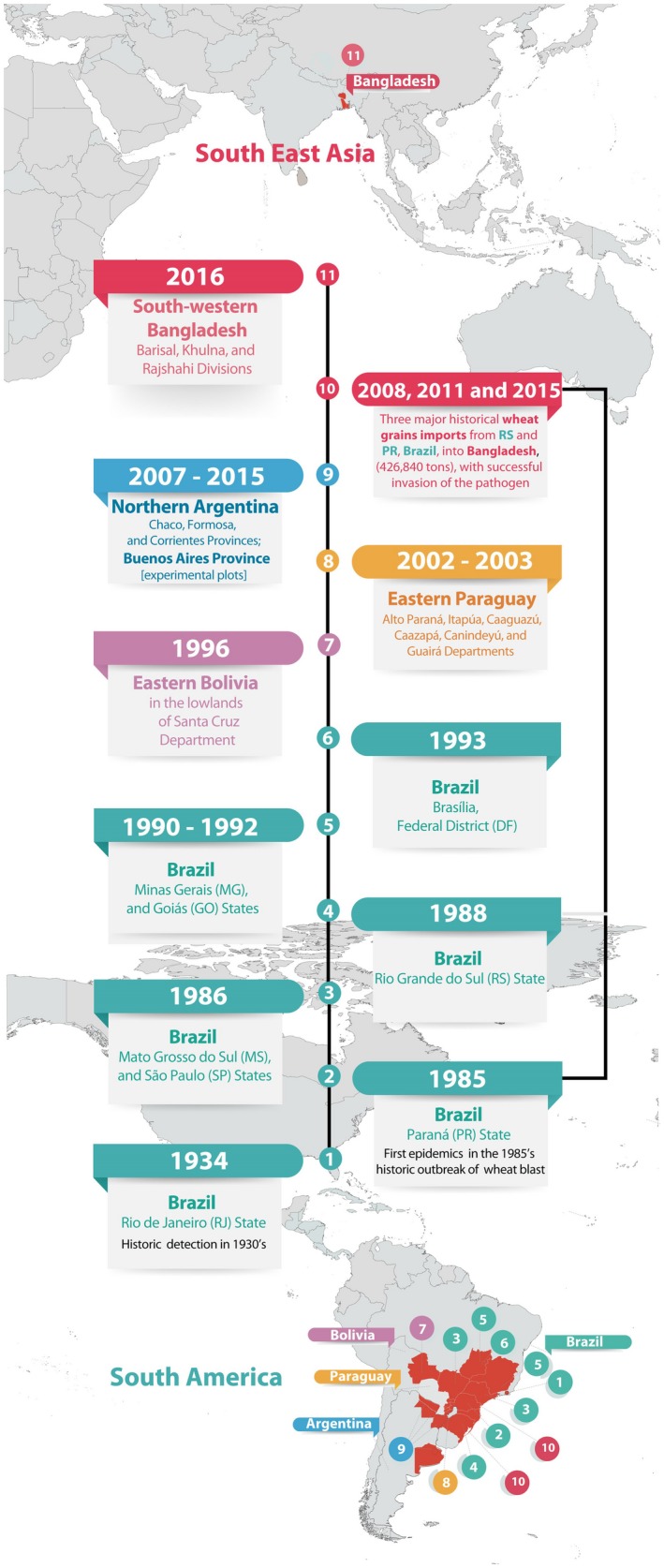
Timeline of events for the spread of wheat blast, from its emergence in South America to its invasion into South‐East Asia. Regions with confirmed wheat blast are highlighted in red. [Colour figure can be viewed at wileyonlinelibrary.com]

Wheat blast disease was first discovered in the state of Paraná, Brazil in 1985 (Igarashi *et al*., 1986); it was found in the neighbouring states of São Paulo and Mato Grosso do Sul in 1986 (Goulart *et al*., 1990), followed by Rio Grande do Sul in 1987 (Igarashi, 1991) (Fig. 1). Since then, wheat blast has become a major disease across central and southern Brazil and is now well established in South America. The disease was not widespread in Brazil before the 1985 epidemic (Igarashi, 1991; Igarashi *et al*., 1986), although a previous description from 1936 (Puttemans, 1936) suggests that wheat blast may already have been present in the state of Rio de Janeiro (Fig. 1).

Wheat blast is caused by a hemibiotrophic Ascomycete in the *Pyricularia *species complex. The correct name for the *Pyricularia* lineage(s) causing wheat blast is currently under debate (Ceresini *et al*., 2018; Gladieux *et al*., 2018). In this review, we refer to the collection of lineages found to be primarily associated with wheat as *Pyricularia* *graminis‐tritici* (*Pygt*), following Castroagudín *et al*. (2016). We address the taxonomic debate about the species status of *Pygt* later in this review. After its origin in Paraná, the pathogen followed the agricultural expansion to the warm Cerrado areas of central‐western Brazil, arriving in Minas Gerais in 1990 (Lima, 2004), Goiás in 1992 (Prabhu *et al*., 1992) and Brasília in 1993 (Anjos *et al*., 1996), spreading about 1200 km north from its origin. *Pygt *also invaded new wheat agroecosystems located 1700 km to the north‐west of Paraná, arriving in Bolivia in 1996 (Barea and Toledo, 1996) and eastern Paraguay in 2002 (Viedma *et al*., 2010; Viedma and Morel, 2002). It also spread to cooler regions 1200 km south‐west of Paraná, reaching Chaco and Corrientes provinces in Argentina in 2007 (Alberione *et al*., 2008; Cabrera and Gutiérres, 2007; Perelló *et al*., 2015) (Fig. 1).

Seed‐borne inoculum probably facilitated the long‐distance dispersal of *Pygt* and allowed it to invade other agroecosystems in South America and now South‐East Asia (Gomes *et al*., 2017; Goulart and Paiva, 1992a; Goulart *et al*., 1995). Despite the risk of introducing *Pygt* into their local agroecosystems, 65 countries imported Brazilian wheat or mixtures of wheat and rye between January 2006 and September 2017, with quantities as high as 1.14 million tons of seeds or grain (Ceresini *et al*., 2018; Government of Brazil, 2017). A series of imports between 2008 and 2015, totalling more than 425 000 tons of possibly contaminated mixtures of wheat and rye grain harvested from Brazilian wheat blast epidemic areas, preceded the emergence of *Pygt* in Bangladesh in 2016 (Islam *et al*., 2016).

The invasion of wheat blast into Bangladesh in 2016 (Callaway, 2016; Islam *et al*., 2016) and its possible spread into India in 2017 (Government of India, 2016; Press Trust of India, 2017) brought wheat blast to the attention of Asian governments and the international community of plant pathologists, exposing an urgent need to develop strategies to contain the spread of this destructive pathogen (Islam *et al*., 2016; Malaker *et al*., 2016; McDonald and Stukenbrock, 2016; Sadat and Choi, 2017; Saharan *et al*., 2016; Sharma, 2017; Singh *et al*., 2016). A suitable climate, coupled with highly susceptible cultivars, could lead to severe outbreaks of wheat blast if it spreads further into India’s north‐eastern plains and Pakistan. This could have serious consequences for regional food security (Government of India, 2016; Saharan *et al*., 2016).

## The Origin of the Wheat Blast Pathogen

Early studies conducted in the 1990s indicated a non‐rice origin for the wheat blast pathogen (Urashima *et al*., 1993). The findings of cross‐infection and inter‐fertility between fungal strains originating from different grass hosts occurring near wheat fields suggested that non‐rice hosts played a key role in the emergence of wheat blast in Brazil (Castroagudín *et al*., 2016; Pereira *et al*., 2014; Urashima *et al*., 1993). Isolates of *Pygt *found on wheat can infect a wide range of invasive, native and cultivated grass hosts from the tribes Hordeae, Festuceae, Aveneae, Chlorideae, Agrosteae and Paniceae (Urashima *et al*., 1993). Physical proximity between wheat and other grass species under natural field conditions was proposed to facilitate genetic exchange among the *Pyricularia *populations infecting different hosts, enabling host shifts (Stukenbrock and McDonald, 2008). Field experiments are needed to test these hypotheses and to provide a better understanding of the role played by non‐wheat hosts in the epidemiology of wheat blast.

Evidence supporting the hypothesis that wheat blast emerged via a host shift from a *Pyricularia* population infecting *Lolium* came from analyses of genetic variation in the avirulence genes *PWT3 *and *PWT4 *(Inoue *et al*., 2017). In this model, *Lolium‐*derived isolates carrying the Ao avirulence allele at the *PWT3 *locus infected a susceptible wheat cultivar carrying the *rwt3 *allele conditioning susceptibility, with the 1980s wheat blast outbreak in Brazil enabled by the widespread cultivation of susceptible wheat cultivars carrying *rwt3*. Later, selection on the less common *Rwt3 *wheat varieties favoured the emergence of pathogen strains with non‐functional *PWT3* alleles. The model proposes that these *pwt3 *strains became the epidemic wheat blast population prevalent in South America (Inoue *et al*., 2017). Although this model made use of available data, the study did not include any *Lolium*‐associated populations of the pathogen from wheat blast endemic areas in Brazil, and did not consider that the association of *Pyricularia* with *Lolium* was reported 7 years after the Brazilian wheat blast outbreak (Igarashi *et al*., 1986; Nunes *et al*., 2002; Urashima *et al*., 1993). This model should be further tested using extensive collections of *Lolium*‐derived *Pygt* from Brazil to compare levels of gene and genotype diversity with the wheat‐infecting population and to determine whether the sequences of *PWT3 *and *PWT4* in the Brazilian *Lolium*‐infecting population are consistent with this model.

**Figure 2:**
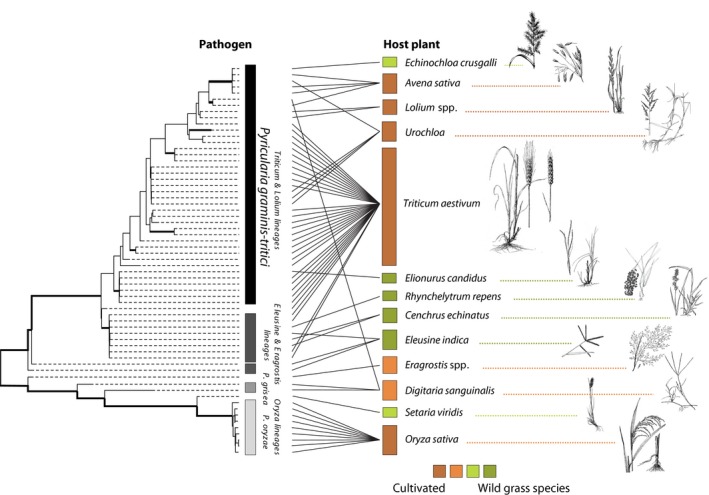
Maximum likelihood phylogenetic tree of *Pyricularia* lineages based on 28 427 genome‐wide single nucleotide polymorphisms. Bold lines indicate strong (100%) bootstrap support. The gradients across wild and cultivated host plant species are indicated by different colours. Plant drawings were adapted from original images obtained from the USDA Natural Resources Conservation Service ‐ NRCS (2018). [Colour figure can be viewed at wileyonlinelibrary.com]

Population genomic analyses, including 36 *Pygt *strains originating from many different hosts (including *Lolium*) and 59 strains of other *Pyricularia *species, could not determine whether the wheat blast pathogen in Brazil had a single or multiple origins. The absence of strict host specialization amongst the major *Pygt* subclades indicates that the capacity to infect wheat may have originated multiple times (Castroagudín *et al*., 2017; Fig. 2). This finding is consistent with the emergence of wheat blast in Brazil through a series of host shifts from populations of *Pygt* infecting native or invasive grass species growing near wheat fields (Castroagudín *et al*., 2015, 2017; Fig. 2). Supporting this hypothesis was the discovery of contemporary gene flow between the wheat‐infecting *Pygt* population and the *Pygt *population infecting other grass species, with the notable exception of the rice‐derived population *P. oryzae *(Castroagudín *et al*., 2017). Despite the lack of evidence for extensive contemporary gene flow, Gladieux *et al*. (2018) found evidence for historic introgression events amongst *Pygt *and *P. oryzae *populations. Further support came from the finding that the most frequent *Pygt *virulence groups, defined by glasshouse inoculations of single strains onto seedlings and detached ears of seven wheat differential lines (Maciel *et al*., 2014), were shared between the grass‐ and wheat‐infecting populations, suggesting movement of *Pygt* between nearby grasses and wheat crops (Castroagudín *et al*., 2017). Field experiments should be conducted to directly test this hypothesis. The *Pygt* populations found on wheat and other hosts have significantly higher genetic variation than the populations of *P. oryzae *causing rice blast in Brazil (Castroagudín *et al*., 2017; Saleh *et al*., 2012). All of the population genetic and population genomic analyses conducted thus far suggest that the rice blast pathogen may not provide an appropriate model for understanding wheat blast.

The broader host range and apparent genetic diversification of *Pygt *relative to *P. oryzae* raise many questions about the functional role of within‐species polymorphisms. Mutations in the avirulence genes *PWT3 *and *PWT4 *are the only genetic factors that have thus far been associated with adaptation of *Pygt *to its host, allowing the fungus to escape recognition (Inoue *et al*., 2017). However, hundreds of presence/absence gene polymorphisms have been detected both within *Pygt *and between the wheat‐ and rice‐infecting populations (Chiapello *et al*., 2015; Yoshida *et al*., 2016). The gene categories most affected by gene deletion polymorphisms include genes encoding effectors and key secondary metabolites (Yoshida *et al*., 2016). Such genetic variation has been shown to fuel rapid adaptation to new host genotypes in other plant pathogens (Hartmann and Croll, 2017; Hartmann *et al*., 2017). Genomic analyses of the *Pygt *strains sampled from the recent outbreak in Bangladesh in 2016 and 2017 indicated the occurrence of a single clonal genotype, presenting a significant contrast to the high genetic diversity found at the pathogen’s centre of origin in South America (Islam *et al*., 2016). This suggests that *Pygt* experienced a significant bottleneck during its introduction into Bangladesh. Preventing the introduction of additional, sexually compatible strains of the pathogen into Bangladesh will be an important strategy to prevent the Asian *Pygt* population from undergoing the rapid diversification through recombination that happened in South America (Zhan *et al*., 2015).

## The Wheat Blast Pathogen is Highly Diverse and has a Broad Host Range

From the time at which the rice blast pathogen was first named *Pyricularia oryzae* in 1892 (Tosa and Chuma, 2014), several *Pyricularia*‐like isolates associated with blast symptoms on barley (*Hordeum vulgare*), millet (*Eleusine coracana*, *Pennisetum glaucum*, *Setaria italica*), oats (*Avena* *sativa*), perennial ryegrass (*Lolium perenne*), wheat (*Triticum aestivum*) and more than 50 other grass species have been classified under the *P. oryzae* species complex (Couch *et al*., 2005; Couch and Kohn, 2002; Murakami *et al*., 2003; Takabayashi *et al*., 2002; Urashima and Kato, 1998). Based on assumptions with regard to host specificity, mating ability and genetic relatedness, *P. oryzae* was thereafter split into several pathotypes to reflect a limited host range: e.g. the *Avena* pathotype was attributed to isolates pathogenic on oats, the *Eleusine* pathotype to isolates pathogenic on finger millet (*Eleusine coracana*), the *Lolium* pathotype was pathogenic on perennial ryegrass and the *Triticum* pathotype was pathogenic on wheat (Farman, 2002; Kato *et al*., 2000; Oh *et al*., 2002; Tosa *et al*., 2004; Urashima *et al*., 1999). Although it became the status quo, this system of sorting* Pyricularia *isolates based on the assumption of host‐specialized populations does not reflect the current known biology of the blast pathogens, because many *Pyricularia* have been shown to have broader host ranges (Kato *et al*., 2000). For example, isolates of the *Triticum* pathotype can cause blast on barley, oats, rye (*Secale cereale*), signalgrass (*Urochloa brizantha*) and more than 10 other grass species (Castroagudín *et al*., 2016; Urashima *et al*., 1993), isolates of the *Avena* pathotype can infect wheat (Oh *et al*., 2002) and isolates of the *Lolium* pathotype can also infect wheat (Farman *et al*., 2017; Tosa *et al*., 2004). Further complicating the definition of species boundaries in this species complex is that different sets of strains, typically coming from different hosts, geographically distant locations and often collected many years apart, are mixed together into a single analysis oriented around a single set of genetic markers or phenotypes, making it difficult to compare and unify results coming from different analyses. We believe that new population genomic studies that bring together a representative set of *Pyricularia* strains collected from different hosts in sympatry (i.e. from the same geographical region and in the same time frame) will clarify the association between host range and the possibility of recent speciation.

Since 2010, a series of comprehensive phylogenetic studies have revisited the relationships among *Pyricularia *and* Pyricularia*‐like species, leading to substantial changes in the order Magnaporthales (Choi *et al*., 2013; Hirata *et al*., 2007; Klaubauf *et al*., 2014; Luo and Zhang, 2013; Murata *et al*., 2014). Taxonomists have proposed a definitive name change from *Magnaporthe *spp. to *Pyricularia *spp. (Klaubauf *et al*., 2014; Luo and Zhang, 2013; Murata *et al*., 2014). Older multi‐gene phylogenetic analyses of highly conserved housekeeping genes already recognized that *P. oryzae* and *P. grisea* were independent phylogenetic species (Couch and Kohn, 2002). Newer phylogenetic analyses based on 859 067 single nucleotide polymorphisms (SNPs) distributed across entire genomes have indicated that *Pyricularia* isolates associated with *Triticum* and *Avena* form a single monophyletic clade clearly distinct from the *P. oryzae *clade (Yoshida *et al*., 2016). These results were coherent with earlier phylogenomic analyses (Chiapello *et al*., 2015) and suggest that *P. oryzae* may not provide a good model for understanding the biology of the wheat blast pathogen.

A recent study based on a multi‐gene phylogeny for 128 isolates of *Pyricularia *spp*. *sampled from sympatric populations associated with rice, wheat and grasses growing near wheat fields distinguished the new species *P. graminis‐tritici* associated with wheat and several other hosts (Castroagudín *et al*., 2016)*. *This study, which used the same 10 housekeeping genes that separated *P. oryzae* from *P. grisea* (Couch and Kohn, 2002), identified two *Pyricularia* species associated with wheat blast: *Pygt *and the formerly described *Triticum *pathotype of *P. oryzae* (PoT), which were both independent of the *Oryza* pathotype. The assignment of the new *Pygt *species was challenged by Gladieux *et al*. (2018), although it was also evident in their analysis that the rice‐ and wheat‐associated genotypes were in separate clades and that wheat‐associated genotypes were highly diverse compared with the rice‐associated genotypes. Gladieux *et al*. (2018) presented three arguments against the assignment of a separate species to the wheat‐associated isolates: (1) a lack of phylogenetic concordance for most of the loci analysed; (2) evidence for historic gene flow at specific loci between the genomes of rice‐ and wheat‐associated isolates and; (3) the occurrence of multiple, deeply diverging lineages within the original *P. oryzae*. The concordance of phylogenetic signals at multiple loci is often considered to be a prerequisite for the assignment of a new fungal species (Taylor *et al*., 2000). However, the phylogenetic species recognition system is very conservative and may fail to identify recent speciation events, which may be especially common in plant‐pathogenic fungi associated with agroecosystems (Stukenbrock and McDonald, 2008). Genomic analyses of the early stages of speciation in fungi, plants and animals have shown that the phylogenetic concordance of conserved loci emerges well after reproductive isolation of populations as a result of reduced hybrid fitness (Seehausen *et al*., 2014).

A more recent study including 95 genomic sequences of the wheat blast fungus from Brazil and other *Pyricularia *species supported the designation of *Pygt* as a distinct, highly diverse species with a broad host range (Castroagudín *et al*., 2017; Fig. 2), including strains from the pathotype *Triticum *(PoT) lineage which was placed into a separate clade when only 10 housekeeping genes were used (Castroagudín *et al*., 2016). Hence, the genome‐scale analysis merged the previously described PoT lineage into the *Pygt* species. This study also showed that *Pygt* is capable of causing blast on many other crop species, including barley, oats, perennial ryegrass and signalgrass, as well as native and introduced grass species frequently occurring as weeds in wheat fields (e.g. *Chloris distichophylla*, *Cynodon *spp., *Digitaria insularis*, *Equinochloa crusgalli*, *Panicum* *maximum*, *Rhynchelytrum repens *and* Sorghum sudanense*) (Castroagudín *et al*., 2017; Fig. 2). This new study has three important implications: (1) *Pygt* is not a wheat‐specialized pathogen; (2) the hypothesis of grass‐specific populations for the overall *P. oryzae* species complex is falsified; and (3) *P. oryzae* may not provide a suitable model for understanding the biology of *Pygt*.

The recognition of *Pygt *as a distinct species has important implications for quarantine regulations worldwide (Castroagudín *et al*., 2017). A lack of recognition that the formerly geographically restricted wheat blast pathogen *Pygt* was not the same species as the globally distributed rice blast pathogen *P. oryzae* may explain in part the lack of phytosanitation screens that led to the introduction of* Pygt *into Bangladesh (Government of Brazil, 2017; Ceresini *et al*., 2018). It is possible that local officials assumed that the wheat blast pathogen *Pygt* was the same species as the rice blast pathogen *P. oryzae*, which was already a well‐established pathogen in Bangladesh (Government of the People’s Republic of Bangladesh, 1989, 2011; Khan *et al*., 2016). Regardless of the root cause(s) of the introduction of wheat blast into Asia, it is clear that improved quarantines should be implemented to limit the further spread of *Pygt* into new regions.

## Wheat Blast has Limited the Expansion of Wheat Production in Brazil

**Figure 3:**
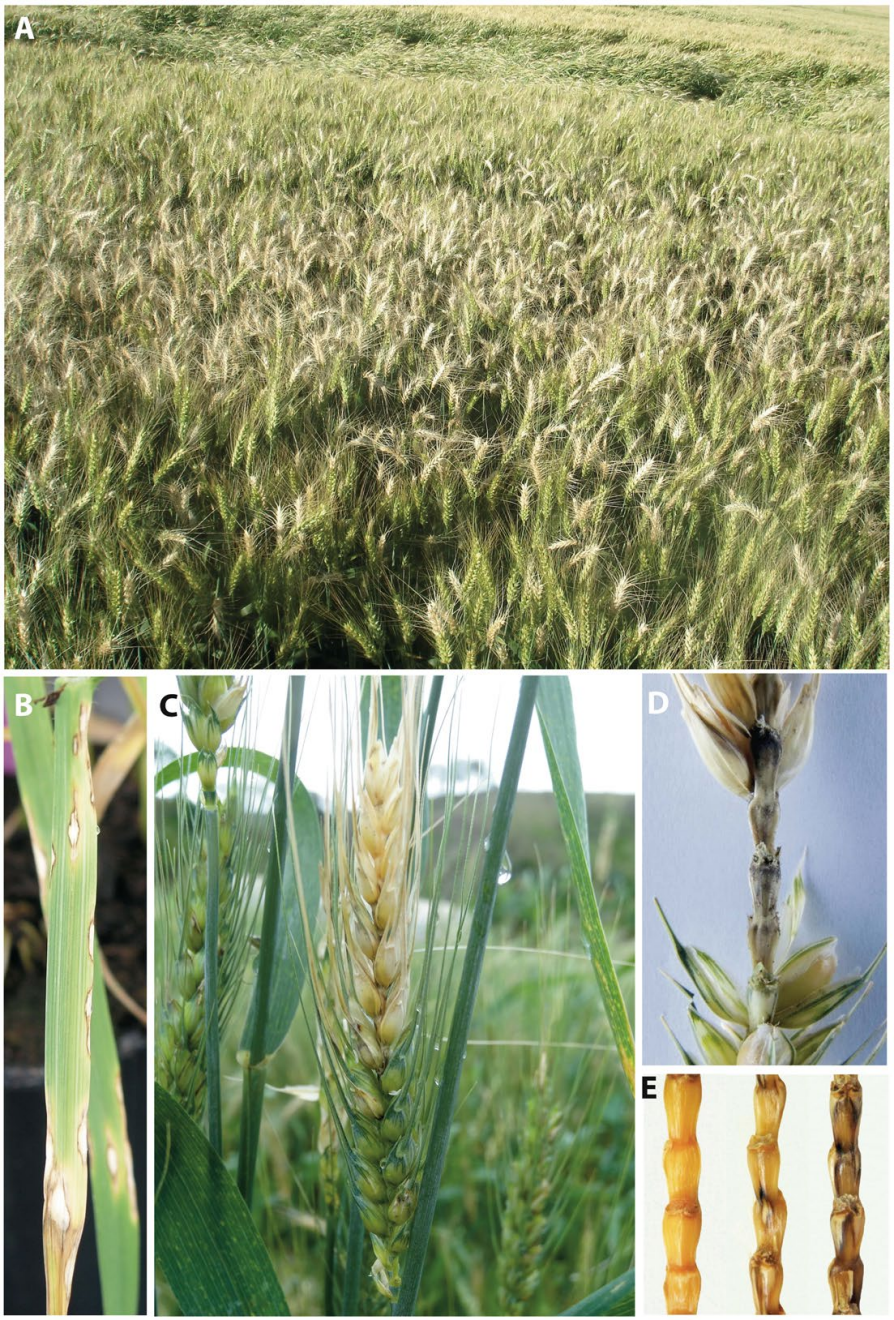
Symptoms associated with wheat blast, including severe head infection in an irrigated wheat field in the Cerrado (A), and infections on leaves (B), spikes (C) and rachis (D, E). [Colour figure can be viewed at wileyonlinelibrary.com]

Wheat blast has limited the expansion of wheat cropping in Brazil. An analysis of the history of wheat production in the state of Mato Grosso do Sul illustrates its impact. In 1987, the wheat crop reached a peak of 428 000 hectares in Mato Grosso do Sul. Mainly as a result of wheat blast epidemics (Fig. 3A), but also in response to frequent droughts and falling commodity prices, wheat fields are now rare in this state, dropping by 95% to less than 20 000 ha in 2016 (Companhia Nacional de Abastecimento ‐ CONAB, 2017).

**Figure 4:**
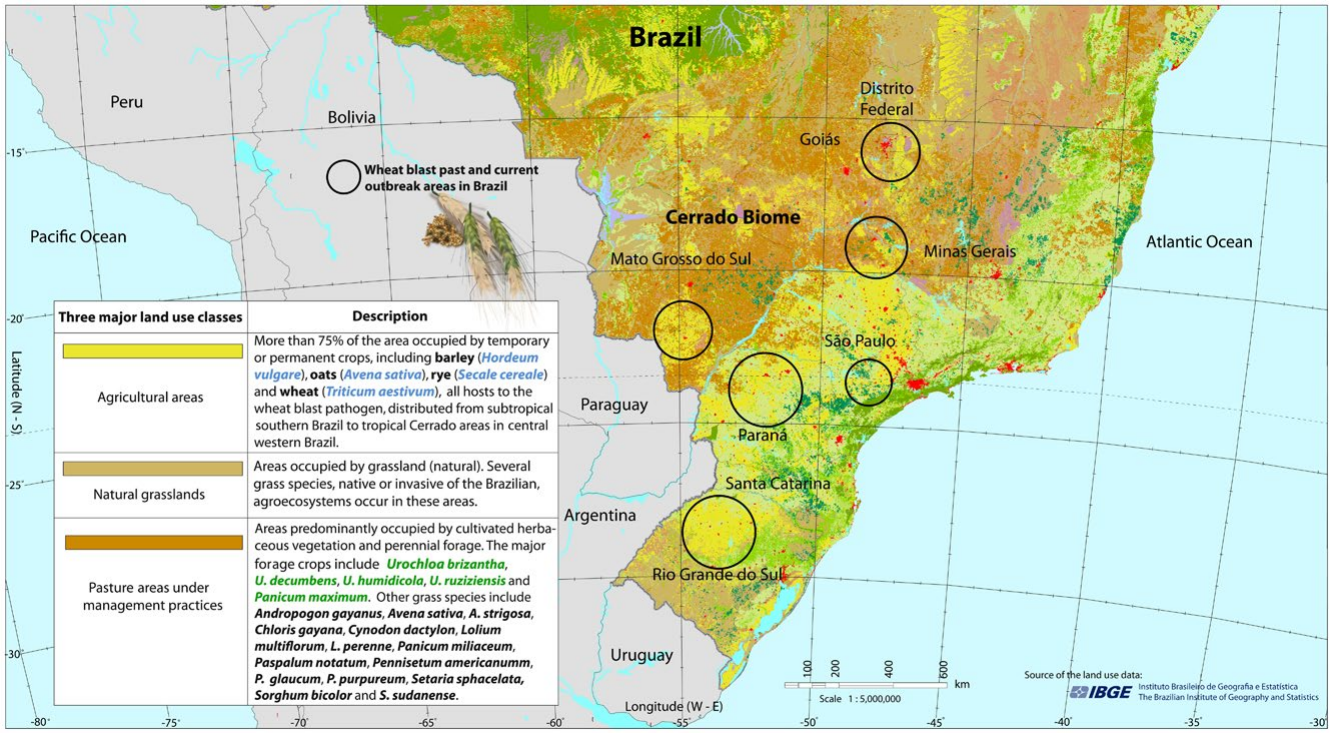
A map of Brazil showing the distribution of the three major land use categories and their overlap with areas affected by wheat blast. Land use data were provided by The Brazilian Institute of Geography and Statistics ‐ IBGE (2016). [Colour figure can be viewed at wileyonlinelibrary.com]

Wheat blast has also prevented the expansion of tropical wheat into the Cerrado region spanning the states of Minas Gerais, Goiás and the Brasília Federal District (Fig. 4). There would be many advantages associated with wheat cultivation in the Brazilian Cerrado. When used in a crop rotation, wheat decreases pathogen inoculum for other crops in the rotation, including the soybean crop that is vital for Brazil’s export economy. The Brazilian Cerrado is closer to the major cities located in central and northern Brazil, resulting in significantly lower transportation costs and making wheat produced in the Cerrado more economically competitive than imported wheat, especially from Argentina (Ceresini *et al*., 2018; Maciel, 2011). However, the fear of yield losses caused by wheat blast leads many growers to avoid cropping tropical wheat (Ceresini *et al*., 2018; Maciel, 2011).

## Biochemical and Physiological Alterations Associated with Wheat Blast

Foliar symptoms of wheat blast are rarely seen before heading and include elliptical to elongate lesions showing white to light brown centres and dark grey to reddish‐brown borders (Fig. 3B). Typical symptoms are observed on the heads when all spikelets above the point of infection in the rachis turn white (Fig. 3C). Symptoms on heads can vary from elliptical lesions with bleached centres on glumes to partial or total spike bleaching, sterility and empty grains (Goulart and Paiva, 1992b; Goulart *et al*., 1990; Urashima *et al*., 2009). Multiple points of infection in the rachis typically spread upwards and downwards from its node (Fig. 3D,E). When grains are produced, wheat blast reduces grain yield and quality, which can render the grain unfit for human consumption (Urashima *et al*., 2009). The shrunken and wrinkled grains commonly found in blasted wheat fields are used mainly for animal feed (Goulart and Paiva, 1992b; Goulart *et al*., 1990; Urashima *et al*., 2009).

Infection of plant leaves and spikes by *Pygt* causes many biochemical and physiological changes. Infected flag leaves show lower ribulose‐1,5‐bisphosphate‐carboxylase/oxygenase (RuBisCO) activity and a reduced RuBisCO activation state (Debona *et al*., 2013), as well as reduced CO_2_ assimilation (Rios *et al*., 2017)*.* Infected leaves also suffer from lower CO_2_ influx as a result of stomatal closure (Aucique‐Pérez *et al*., 2014; Debona *et al*., 2013). Net carbon assimilation (*A*) correlates negatively with CO_2_ internal concentration (*C*_i_), and *A* is negatively correlated with blast severity (Aucique‐Pérez *et al*., 2014; Debona *et al*., 2013). A decrease in light‐saturated *A* (*A*_max_) and the light saturating point (LSP), coupled with an increase in dark respiration (*R*_d_), indicating plant photoinactivation, have been reported in infected leaves (Aucique‐Pérez *et al*., 2017). The photosynthetic machinery of infected leaves is impaired as indicated by the lower values of maximum photosystem II (PSII) quantum efficiency (*F*_v_/*F*_m_), capture efficiency of excitation energy by the open PSII reaction centres (*F*_v_′/*F*_m_′), photochemical quenching coefficient (*q*_P_) and electron transfer rate (ETR).

Flag leaves and grains infected by *Pygt* show lower fructose, glucose and sucrose concentrations and reduced sucrose phosphate synthase (SPS) activity (Rios *et al*., 2017). Infected flag leaves show a high starch concentration because of the down‐regulation of α‐ and β‐amylase genes (Rios *et al*., 2017). In grains obtained from infected spikelets, starch concentration is decreased in response to lower sucrose transport from photosynthetic organs. An increase in the breakdown of starch in grains from infected spikelets has been linked with the expression of α‐ and β‐amylase genes at an early stage of *Pygt* infection (Rios *et al*., 2017). Plants infected by *Pygt* during reproduction show alterations in the metabolism of organic acids and amino acids (Rios *et al*., 2017). The concentrations of amino acids derived from glycolytic intermediates (e.g. alanine, cysteine, phenylalanine and valine), as well as arginine, cysteine, histidine, methionine, proline and tryptophan, are higher in infected leaves. Fructose, glucose, sucrose and starch concentrations are dramatically reduced in grains obtained from infected spikelets (Rios *et al*., 2017).

High concentrations of hydrogen peroxide (H_2_O_2_) and superoxide anion radical (O_2_^•^), and a reduction in lipid peroxidation, are essential for leaf and spike resistance to blast, especially on cultivars with a high level of partial resistance (Debona *et al*., 2012; Silva, 2017). Higher activities of ascorbate peroxidases (APXs), catalases (CATs), glutathione‐*S*‐transferases (GSTs), glutathione reductases (GRs), peroxidases (POXs) and superoxide dismutases (SODs) have been associated with the high level of basal resistance of some cultivars (Debona *et al*., 2012). Blast severity decreases on leaves of plants sprayed with picolinic acid because of a rapid response of antioxidant metabolism (higher APX, GST, POX and SOD activities) in the removal of reactive oxygen species (Aucique‐Pérez, 2016). Polyphenoloxidase activity on infected flag leaves is high regardless of the basal level of resistance of the cultivar. Phenylalanine ammonia‐lyase activity and the concentrations of phenolics and lignin are higher on more resistant cultivars (Silva, 2017). Chitinase and β‐1,3‐glucanase activities increase in response to *Pygt* infection on leaves and spikes of cultivars with higher resistance (Silva, 2017).

## Population Genetics of *PYGT* and Epidemiology of Wheat Blast

Contemporary populations of *Pygt *collected from across the wheat‐growing regions of Brazil in 2012 and 2013 (including more than 500 strains taken from wheat and grass species growing in or nearby infected fields) contained high genetic and virulence diversity, including 198 multilocus microsatellite genotypes (MLMGs) and 25 virulence groups (Castroagudín *et al*., 2017). These populations exhibited a mixed reproductive system in which cycles of sexual reproduction generated novel genotypes that could be selected by the local environment, enabling amplification and dispersal of well‐adapted clones via asexual reproduction (Castroagudín *et al*., 2017). A similar genetic structure was observed in populations of *Pygt* previously sampled from central‐southern Brazil (Maciel *et al*., 2014). Morphological evidence supporting the hypothesis of sexual reproduction included the detection of proto‐perithecia and perithecia forming on senescing stems of wheat and other grasses under experimental conditions, suggesting that sexual reproduction can occur on crop residues or within senescent tissues of alternative hosts during the saprotrophic phase of the disease cycle (Castroagudín *et al*., 2017; Moreira *et al*., 2015). An important area for future research will be to identify the main hosts, tissues and environmental conditions that support the development of the sexual cycle in nature. The complete development of perithecia was observed on *Phalaris canariensis* (canarygrass), indicating that it is a promising candidate for the observation of the teleomorph in the field.

Given that *Pygt *isolates are capable of infecting wheat and other grasses, and can move back and forth between hosts, we hypothesize that sexual recombination may occur preferentially on non‐wheat hosts with the resulting ascospores and/or conidia infecting nearby wheat fields, giving rise to the highly diverse *Pygt *population detected on wheat. This scenario is supported by previous reports of gene flow amongst wheat‐ and other Poaceae‐adapted populations of *Pygt* (Castroagudín *et al*., 2017), the sharing of genotypes and virulence phenotypes between the host groups (Castroagudín *et al*., 2017), the cross‐infection and inter‐fertility between isolates from other poaceous hosts and wheat (Bruno and Urashima, 2001; Galbieri and Urashima, 2008; Urashima *et al*., 1993), and the occurrence of gametic equilibrium among neutral genetic markers (Castroagudín *et al*., 2017), consistent with sexual recombination in most populations, including those from non‐wheat hosts (Castroagudín *et al*., 2017).

The populations of *Pygt* from other grass species included strains isolated from signal grass (*U. brizantha*) (Castroagudín *et al*., 2017). As signal grass is an extensively grown pasture grass occupying more than 90 million hectares in Brazil (Jank *et al*., 2014), and is frequently found alongside wheat fields (The Brazilian Institute of Geography and Statistics ‐ IBGE, 2016; Fig. 4), it may play an important role as a key inoculum reservoir for wheat blast, providing a spatial and temporal link connecting wheat fields across central and western Brazil. A high degree of gene flow across a spatial scale of more than 2000 km was found in Brazil (Castroagudín *et al*., 2017). This high gene flow, which would maintain many alleles and reduce the impact of genetic drift, could reflect efficient wind dispersal of the pathogen’s conidia or ascospores over short distances (Urashima *et al*., 2007), in addition to long‐distance dispersal via infected seeds of wheat and *Urochloa *(Gomes *et al*., 2017; Goulart *et al*., 1995; Goulart and Paiva, 1993a, 1993b). Overall, these findings suggest that non‐wheat hosts may play an important role in the epidemiology of wheat blast, further complicating control efforts. There is an urgent need for field experiments to better understand the epidemiology of wheat blast, aiming to elucidate the relative importance of infected seeds, conidia and ascospores, coming from both wheat and non‐wheat hosts, as sources of primary and secondary inoculum.

One of the important findings on the population genetics of *Pygt *was the discovery of a super‐race, named virulence group A, which caused blast on the entire tested panel of wheat and barley cultivars (Castroagudín *et al*., 2017; Ceresini *et al*., 2018). Virulence group A was not a clone; this phenotype was found in many different *Pygt* genetic backgrounds. This suggests that the virulence group A phenotype may represent the loss of a major avirulence gene that is now segregating in many different genetic backgrounds as a result of recombination. It was detected at a higher frequency on Brazilian wheat, but was also found on several grass species invading wheat fields, including *Avena* *sativa*, *Cenchrus echinatus*, *Chloris distichophylla*, *Cynodon *spp., *Echinochloa crusgalli*, *Digitaria insularis*, *D. sanguinalis*, *Eleusine indica*, *Eragrostis plana*, *Panicum* *maximum*, *Rhynchelytrum repens*, *Sorghum sudanense *and *U. brizantha* (Castroagudín *et al*., 2017; Ceresini *et al*., 2018). Other common virulence groups were also shared between the grass‐ and wheat‐infecting *Pygt *populations, providing additional evidence for the movement of *Pygt* between wheat fields and nearby grasses.

## Strategies for the Management of Wheat Blast

### Reinforcing quarantine and bio‐safety rules in wheat blast‐free regions

Because wheat blast has now escaped from its endemic areas in South America and is causing epidemics in South‐East Asia, the primary global concern is to prevent additional spread of the pathogen to disease‐free countries and to avoid potential outbreaks in new regions. As infected seeds can spread the pathogen over long distances (Cruz and Valent, 2017; Goulart and Paiva, 1990; Silva *et al*., 2009; Urashima *et al*., 1999), the strengthening of quarantine and seed trading laws will provide the best course of action to prevent the further spread of wheat blast (Mezzalama, 2016; Sadat and Choi, 2017; Singh, 2017; Valent *et al*., 2013). Trade in wheat seeds from the wheat blast endemic areas in Latin America and South‐East Asia should be strictly regulated, if not totally prohibited. A globally organized awareness programme should advise quarantine regulators worldwide to implement appropriate rules to address the threat posed by the spread of wheat blast. Local country officials should more strictly enforce laws prohibiting the re‐direction of wheat grains imported to make flour into the local wheat seed industry. The introduction of wheat blast into Bangladesh appeared to follow the latter path (Ceresini *et al*., 2018; Islam *et al*., 2016; Sadat and Choi, 2017).

### Management strategies in wheat blast endemic areas

Integrated disease management (IDM) strategies will be needed to minimize crop losses without impacting environmental sustainability (Mehta, 2014). The implementation of IDM tactics should be organized locally, taking into account the particular conditions of each affected region (Mehta, 2014). These tactics should be based on a knowledge of the pathogen’s population biology and epidemiology, including its disease cycle, survival strategy, means of dispersal, host range, main reproductive mode(s) and the weather conditions most conducive for disease development. Unfortunately, much of this knowledge is still missing.

#### Crop rotations or other cultural and sanitary practices

Weedy grass hosts appear to play a key role as bridges between wheat fields and cropping seasons and as sources of primary and/or secondary inoculum. Reducing the population of known weed hosts within and nearby wheat fields may be difficult to implement, but could reduce local *Pygt *inoculum (Mehta, 2014). It appears that *Pygt* can survive and produce sexual fruiting bodies (perithecia) on crop residues between wheat cropping seasons (Castroagudín *et al*., 2017; Urashima and Kato, 1998). If minimum tillage is not essential for soil conservation purposes, deep ploughing could decrease the initial *Pygt* inoculum on crop stubble (Igarashi, 1991; International Maize and Wheat Improvement Center ‐ CIMMYT Wheat Program, 2016). Following the introduction of wheat blast into South‐East Asia, local officials advised the elimination of crop residues from wheat, barley, millet and oats, as well as the eradication of invasive grass hosts, including *Brachiaria *spp.,* Cenchrus *spp.,* Chloris* spp*., Digitaria *spp*.*,* Echinochloa *spp., *Eleusine indica *and* Lolium *spp*.* (Government of India, 2016; Plantwise, 2016; Sadat and Choi, 2017). This management strategy may not be cost‐effective for small farmers or appropriate for regions in which soil conservation agriculture is needed (International Maize and Wheat Improvement Center ‐ CIMMYT Wheat Program, 2016). Crop rotation can be a useful option for the management of wheat blast if non‐grass crops, such as soybean and common vetch (Santos *et al*., 2000), are available. In South‐East Asia, jute (*Corchorus olitorius*) cultivation could be an option for crop rotation (Government of India, 2016). Altering the sowing date to prevent an overlap between flowering or grain filling stages and blast‐conducive periods characterized by high temperatures, rain and high relative humidity is an effective cultural practice to manage wheat blast in South America (Coelho *et al*., 2016; Mehta, 2014; Mehta *et al*., 1992). Field experiments designed to dissect the epidemiology of wheat blast will be needed to determine which of these strategies will have the greatest impact.

#### Certified healthy seeds and improved detection methods for seed‐borne inoculum

Because wheat blast can be seed‐borne and seed‐transmitted (Goulart and Paiva, 1990; Martins *et al*., 2004), infected seeds are thought to be the primary source of inoculum for long‐distance dispersal (Cruz and Valent, 2017; Goulart and Paiva, 1990; Silva *et al*., 2009; Urashima *et al*., 1999, 2009). Spores of *Pygt* are able to survive and remain infectious for up to 2 years on both the surface and inside seeds. Even apparently healthy seeds harvested from infected fields can carry fungal spores (Reis *et al*., 1995). Consequently, the use of certified pathogen‐free seeds, or fungicide‐treated wheat seeds, should be mandatory for both internal seed markets and for export from countries with wheat blast (Toledo, 2015). In Brazil, wheat seed fungicide treatments were mandated by law (Mehta, 2014), but, since the 1970s, the legislation has been relaxed and seed health testing has been neglected (Mehta, 2014). This decreased vigilance facilitated the dispersal of seed‐borne *Pygt *inoculum across Brazil’s wheat cropping areas, as well as into Bolivia and Paraguay, through frequent imports of Brazilian wheat seed used for sowing (Ceresini *et al*., 2018).

Accurate methods for the detection of *Pygt* in asymptomatic seeds will be needed to limit or prevent the spread of the pathogen into disease‐free areas. Healthy wheat seed will also decrease the initial inoculum in wheat blast endemic areas (Akhtar *et al*., 2016; Mezzalama, 2016). Ideal detection methods should combine several specific molecular markers as targets and use template DNA extracted directly from potentially infested seed lots (Chen *et al*., 2016; Pieck *et al*., 2016). A recent report has illustrated the current lack of robust molecular tools that can detect infected seeds (Gupta *et al*., 2018), and has highlighted the need to develop improved detection methods.

#### Fungicide seed treatment

The only fungicide labelled for seed treatment against wheat blast in Brazil is the demethylation inhibitor (DMI) difenoconazole. The eradicant fungicide dimethyldithiocarbamate thiram, which is labelled for the control of other seed‐borne wheat pathogens, is also effective against *Pygt *(Bockus *et al*., 2015; Goulart and Paiva, 1991).

#### Disease forecasting based on weather conditions

The optimum weather conditions for wheat blast development include long and frequent periods of leaf wetness (24–40 h), coupled with high temperatures (25–30 °C) (Cardoso *et al*., 2008). In Brazil, a predictive model for wheat blast outbreaks based on daily climatic data, named *Sisalert* (*Plant Disease Epidemic Risk Prediction System*), was developed to calculate the risk of an epidemic (available at: https://dev.sisalert.com.br/monitoramento/?page_id=14; Fernandes *et al*., 2017; Nicolau *et al*., 2016). In the USA, a climatic model adapted from this Brazilian model indicated that the weather conditions were favourable to wheat blast in 25% of the winter wheat cropping regions, with suitable conditions for wheat blast outbreaks in 70% of the years for Louisiana, Mississippi and Florida (Cruz *et al*., 2016). Similar predictive models should be developed for other countries facing wheat blast outbreaks, including Bolivia, Paraguay, Bangladesh and India. The major advantage of using a model like *Sisalert* based on the automated collection of weather data is to provide a warning of an imminent risk of wheat blast infection (West *et al*., 2017; West and Kimber, 2015). This alert enables real‐time decision‐making for fungicide applications.

#### Breeding for wheat blast resistance

The breeding of wheat cultivars for improved blast resistance has not been very successful despite constant efforts over the last 30 years (Cruz *et al*., 2010; Maciel *et al*., 2008; Prestes *et al*., 2007; Urashima *et al*., 2004). With the realization that other grass species, such as *Urochloa* or *Lolium*, may play a key role in wheat blast epidemiology, it may be time to consider breeding for resistance to *Pygt* in these other grass hosts. Resistance to wheat blast is not stable because resistant varieties become susceptible when they are deployed across different geographical locations spanning a 2800‐km transect (Urashima *et al*., 2004). Initially, the unstable resistance was thought to result from cultivar‐by‐environment interactions that differed across Brazil (Castroagudín *et al*., 2016; Duveiller *et al*., 2010; Maciel *et al*., 2014). Today, it seems more likely that the instability of blast resistance reflects a breakdown of resistance genes underlying gene‐for‐gene (GFG) interactions, with new races of the pathogen emerging in different regions to render the deployed resistance ineffective.

Extensive phenotyping of 173 *Pygt* strains encompassing 80 unique MLMGs from six geographical regions in Brazil (Castroagudín *et al*., 2017) differentiated 25 seedling virulence groups (SVGs), named SVGs A–Y (Ceresini *et al*., 2018). The predominant group was SVG L, representing 47% of the isolates tested, whereas SVG A was the second most frequent group, found in 13% of the isolates tested. Many of the wheat cultivars used as differentials lost their resistance to wheat blast in the heading stage, corroborating earlier findings (Urashima and Kato, 1998). The same 173 isolates fell into nine head virulence groups (HVGs) when virulence was assessed on detached, mature wheat heads (Castroagudín *et al*., 2017; Ceresini *et al*., 2018). HVG A was the predominant virulence group, found in 78% of isolates, followed by HVG B, found in 14% of isolates. These two HVGs were found in all geographical regions, including the populations sampled from non‐wheat hosts in Mato Grosso do Sul and Paraná. HVG A caused blast on the entire set of differential cultivars, suggesting its designation as a ‘super‐race’. HVG A was also found on the non‐wheat hosts *Avena* *sativa*, *Cenchrus echinatus*, *Chloris distichophylla*, *Cynodon *spp., *Digitaria insularis*, *D. sanguinalis*, *Echinochloa crusgalli*, *Eleusine indica*, *Eragrostis plana*, *Panicum* *maximum*, *Rhynchelytrum repens*, *Sorghum sudanense* and *U. brizantha*.

Few sources of resistance to wheat blast have been identified in Brazil until now. Amongst these sources, the Brazilian wheat cultivars BRS 229 and BR 18‐Terena have been regularly used in breeding programmes because of their higher levels of field resistance to head blast (Brunetta *et al*., 2006; Goulart *et al*., 1991; Goulart and Paiva, 1992b; Sousa, 2002). However, these resistant cultivars have shown susceptibility in some environments, probably as a result of the emergence of new *Pygt* races in these areas (Urashima *et al*., 2001, 2005). Therefore, the identification of new sources of resistance is crucial.

A potential source of resistance was identified in *Aegilops tauschii* (syn. *Aegilops squarrosa*) (Urashima and Kato, 1994) in 1994. The bread wheat cultivar Renan (Hanzalová *et al*., 2007) and the barley lineage CGN02857 (a barley accession obtained in East Africa and maintained by the CGN germplasm collection at Wageningen University and Research Centre), both derived from crosses with *A. tauschii*, were considered to be promising sources of wheat blast resistance.

Recently, the 2NS/AS chromosomal translocation from *A. ventricosa* was associated with resistance to wheat blast (Cruz *et al*., 2016). Compared with accessions lacking 2NS/AS, near‐isogenic lines of both spring and winter wheat carrying the segment showed a 64%–81% reduction in head blast severity in field trials conducted under natural epidemics in Bolivia. Cultivars derived from the CIMMYT line Milan, which possesses the 2NS/AS translocation, also showed high levels of blast resistance under field conditions (Cruz *et al*., 2016). Other 2NS/AS carriers derived from Milan, such as Canindé I, CD 116 and Sausal ClAT, have been widely deployed in South America, but the stability of this source of resistance remains unclear (Kohli *et al*., 2011). There is evidence that the 2NS/AS resistance present in the bread wheat cultivar Renan can be overcome by three of the nine *Pygt *virulence groups identified in Brazil, including the super‐race HVG A (Castroagudín *et al*., 2017; Ceresini *et al*., 2018). Consequently, additional sources of resistance should be sought and developed.

Four resistance genes (*Rmg2*, *Rmg3*, *Rmg7*, *Rmg8*) have been identified in common wheat (*T. aestivum*) and in tetraploid emmer wheat (*T. dicoccum*) (Anh *et al*., 2015; Tagle *et al*., 2015; Zhan *et al*., 2008). These four resistance genes function against the Br48 strain of *Pygt* that was collected in 1991. The sources of these resistance genes include *T. aestivum* cultivar Thatcher (carrier of *Rmg2* and *Rmg3*; Zhan *et al*., 2008), *T. dicoccum *lines KU112 (St17), KU120 (St24) and KU122 (St25), carriers of *Rmg7* (Tagle *et al*., 2015), and *T. aestivum* line S615, carrier of *Rmg8* (Anh *et al*., 2015). It is not yet known whether these resistance genes will be effective against contemporary populations of *Pygt*.

Embrapa’s new cultivar Lagoa Vermelha has one of Thatcher’s relatives (NewThatch) in its pedigree. In the 2017 screening of Embrapa wheat genotypes in a wheat blast hotspot in Uberaba, Minas Gerais (MG), Lagoa Vermelha showed high levels of blast resistance during an epidemic year. However, in Patos de Minas, MG, under more tropical conditions, cultivar Thatcher showed high susceptibility to blast (J. L. N. Maciel, personal communication). Interestingly, the *Rmg2* and *Rmg3* genes giving blast resistance in cultivar Thatcher are not effective at high temperatures and do not provide resistance for ear infections (Zhan *et al*., 2008). Consequently, they were probably not associated with the levels of resistance found in Lagoa Vermelha. Instead, it is more plausible that cultivar Lagoa Vermelha carries other wheat blast resistance genes.

Progress in breeding for wheat blast resistance will probably be facilitated through the development of uniform sets of host and pathogen differentials that can be used as controls to enable comparisons of results and better coordination of breeding efforts distributed across several locations. Screening of germplasm for wheat blast resistance should include a representative set of the contemporary virulence diversity existing in the wheat blast population across Brazil, including the HVG A super‐race. An optimal field screening for resistance would include several locations across different agroecosystems, including Mato Grosso Sul State, which is a hotspot for the pathogen’s virulence diversity, with the highest number of SVGs (11) and HVGs (6). For countries interested in pre‐emptive breeding for wheat blast resistance, germplasm should be screened for blast resistance in Brazil, where the pathogen is genetically diverse, virulent, endemic and distributed across a temperature cline ranging from the colder regions of Rio Grande do Sul to the warmer Cerrado areas of Goiás (Fig. 4). Three recent examples of this strategy are as follows: a work plan between CIMMYT and the Indian Council of Agricultural Research (ICAR) to test 40 wheat varieties and advanced lines at wheat blast hotspots in Argentina, Bolivia, Brazil and Paraguay (Government of India, 2016); the testing of spring wheat cultivars with and without the 2NS/AS translocation near Quirusillas, in Bolivia (Cruz *et al*., 2016); EMBRAPA Cenargen, at the request of Swiss breeders in cooperation with the Federal University of Lavras, introduced Swiss wheat landraces to test against the most common SVGs and HVGs characterized there (E. Alves, UFLA, personal communication).

#### Fungicide applications on leaves and ears

In South America, fungicides are often used to manage wheat blast even though their efficacy is low, with only a small reduction in blast severity observed on symptomatic spikes in treated fields (Maciel, 2011; Pagani *et al*., 2014). Decreases in crop losses were detected only when mixtures of strobilurin and triazole fungicides were applied early on moderately resistant wheat varieties with low or moderate disease pressure (Pagani *et al*., 2014; Rios *et al*., 2016a, 2016b; Rocha *et al*., 2014). In all cases, the effectiveness of fungicide sprays at early heading and early grain‐filling stages was associated with a decrease in *Pygt* inoculum produced on the lower wheat leaves, with a subsequent reduction in ear infections (Cruz *et al*., 2015).

In South‐East Asia, recommendations for the management of wheat blast include spraying strobilurins combined with triazoles (i.e. trifloxystrobin and tebuconazole) (Government of India, 2016). In Brazil, under high disease pressure, these fungicides resulted in only partial control (Maciel, 2011; Maciel *et al*., 2014), reducing disease severity by 50% (Maciel, 2011). The reduced efficacy of fungicides for the management of wheat blast in Brazil was attributed to many factors, including highly favourable weather conditions coupled with high levels of cultivar susceptibility (Cruz *et al*., 2011), the highly diverse *Pygt *population (Urashima *et al*., 2004), the difficulties of reaching the infection sites on spikelets (Panisson *et al*., 2004) and the inherent inefficacy of some active ingredients (Goulart *et al*., 1996; Maciel, 2011; Pagani *et al*., 2014; Urashima and Kato, 1994). Because *Pygt *has a broad host range, including several invasive grass species on which fungicides are not sprayed (Castroagudín *et al*., 2015; Reis and Casa, 2016), a continuous external source of new inoculum might compromise the efficacy of chemical management (Castroagudín *et al*., 2017). All of these factors should be taken into consideration when fungicides are deployed to manage wheat blast in South‐East Asia.

In Brazil, 28 fungicides were labelled for the management of wheat blast, including 11 triazoles and seven mixtures of quinone outside inhibitors (QoIs) and triazoles (Ministério da Agricultura Pecuária e Abastecimento ‐ MAPA, 2017). These two fungicide groups were used intensively over one to three decades to manage rusts and other foliar diseases of wheat (Debona *et al*., 2009; Navarini and Balardin, 2012; Reis *et al*., 1977; Tormen *et al*., 2013), and it is possible that their use to control other diseases inadvertently selected for resistance in the associated *Pygt* populations, explaining their low efficacy against wheat blast. In fact, resistance to both QoI (azoxystrobin and pyraclostrobin) and triazole (tebuconazole and epoxiconazole) fungicides was found to be pervasive in populations of the pathogen across the major wheat cropping areas from central western to southern Brazil (Castroagudín *et al*., 2015; Ceresini *et al*., 2018; Oliveira *et al*., 2015; Poloni, 2016). All contemporary populations of *Pygt* sampled in 2012 and 2013 showed high resistance to azoxystrobin, pyraclostrobin and to both epoxiconazole and tebuconazole, with half maximal effective concentration (EC_50_) values at least 30–200 times higher than the wild‐type isolates (Ceresini *et al*., 2018). Mutations in the *cytb* and *Cyp51A *genes were associated with higher levels of resistance to strobilurins and triazoles in these populations (Castroagudín *et al*., 2015; Ceresini *et al*., 2018; Oliveira *et al*., 2015; Poloni, 2016). These observations suggest that these strobilurin and triazole fungicides are not likely to provide long‐term solutions to the management of wheat blast in South‐East Asia.

Recently, five new fungicide formulations were labelled for wheat diseases in Brazil, each containing the second‐generation carboxamide fluxapiroxade (a succinate dehydrogenase inhibitor, SDHI) combined with the QoI pyraclostrobin and/or the DMI epoxiconazole (Ministério da Agricultura Pecuária e Abastecimento ‐ MAPA, 2017), the two fungicide classes to which the Brazilian populations of *Pygt* were already resistant (Poloni, 2016). Because none of these five formulations were labelled specifically for wheat blast, their effectiveness has yet to be determined. An important factor to consider is that the second‐generation SDHIs are also high‐risk fungicides (Ishii and Hollomon, 2015) and resistance could emerge in *Pygt* populations if point mutations occur in any of the three genes encoding the targeted components of the SDH complex (SDH B, C and D) (Amiri *et al*., 2013). When 170 *Pygt* isolates sampled from different locations in 2013 were tested for sensitivity to fluxapyroxad, the majority of the isolates were insensitive, with moderately resistant isolates found in five of the six tested field populations (Casado, 2017). This was surprising because these *Pygt* populations had not yet been exposed to second‐generation SDHIs. No data are yet available regarding mutations in the *sdh* subunits B, C and D that could explain the observed levels of resistance. To reduce the risk that *Pygt* will become resistant to SDHI fungicides, the recommendation is to deploy SDHIs only in mixtures with low‐risk fungicides, such as mancozeb and chlorothalonil (van den Bosch *et al*., 2014), but it is clear that a monitoring programme should be implemented to detect the emergence of known resistance mutations in these genes.

#### Biological control and biofortification

Several biocontrol agents which have been shown to reduce rice blast symptoms under field conditions should be tested for their efficacy against wheat blast. Microbes that control rice blast include *Bacillus methylotrophicus*, *Chaetomium globosum *and *Trichoderma harzianum* (Oliveira *et al*., 2015; Park *et al*., 2005; Singh *et al*., 2012). Some non‐fungicidal chemicals have already been evaluated for their ability to control wheat blast. For example, potassium silicate inhibited fungal growth *in vitro* and potassium phosphate reduced blast severity on three wheat cultivars (Cruz *et al*., 2011). Applications of silicon restricted *Pygt *colonization of wheat leaves by triggering the flavonoid biosynthetic pathway and the deposition of phenolic compounds. Silicon applications also intensified the expression of defence‐related genes (Cruz *et al*., 2011, 2015b, 2015; Silva *et al*., 2015). Treatments with silicon in field experiments increased yields by 26%–92%, whereas phosphite treatments increased yields by 9%–80% (Pagani *et al*., 2014), suggesting promising lines of research for the management of wheat blast with non‐fungicidal chemicals.

## A Forward‐Looking Research Agenda for Wheat Blast

There are many important gaps in our knowledge of wheat blast that will need to be filled in order to develop long‐term management strategies. Although population genetic and genomic studies have provided important insights into the possible origins of *Pygt* and its likely mechanisms of long‐distance dispersal, surprisingly little is understood about wheat blast epidemiology at the field scale. Replicated field experiments are urgently needed to determine the main sources of primary and secondary inoculum and to evaluate the relative contributions of infected seeds, conidia and ascospores to wheat ear infections. Although the population genetic studies suggest that *Urochloa* and other non‐wheat grasses could be important sources of inoculum to fuel wheat blast epidemics, field experiments must be conducted to test this hypothesis. Additional field experiments will be needed to determine the relative contributions of crop residues, infected seeds and non‐wheat grasses to the persistence of *Pygt* between growing seasons and to identify the most important environments for sexual reproduction. The discovery that the *Pygt* population in Bangladesh appears to be a single clone (Islam *et al*., 2016) is inconsistent with the hypothesis that the pathogen was introduced on infected wheat grain colonized by the highly diverse *Pygt* population of Brazil. Although this lack of diversity in the Asian population is good news from the perspective of disease management (less genetic diversity suggests lower evolutionary potential and slower adaptation to fungicide treatments and resistant cultivars), it suggests that much remains to be learned about wheat seed infection.

Equally important will be to identify new sources of wheat blast resistance that are effective and stable. These efforts could be made more efficient by agreeing on a uniform set of host and pathogen differentials to include in screening programmes. Although it seems obvious that the screening for resistance should take place under selection by the highly diverse *Pygt* population in Brazil, it is not obvious what sources of germplasm should be used in the case of a newly emergent disease that did not co‐evolve with its main economic host (wheat), especially given the broad host range of *Pygt*. If non‐wheat hosts, such as *Urochloa*, are shown to be the primary host for the pathogen, with blast disease on wheat mainly collateral damage occurring as a result of inoculum spillover from an epidemic on the non‐wheat host under particular environmental conditions, it may be more appropriate to breed for resistance in the non‐wheat host.

Similarly, if a non‐wheat grass is the primary host of *Pygt*, management strategies may need to focus on the control of the infection in the non‐wheat host in order to prevent spillover into nearby wheat fields. For example, biological control agents or fungicides may be more effective if applied to the primary, non‐wheat host instead of the wheat field. A crop rotation designed to remove *Pygt* inoculum from wheat stubble of previous crops is unlikely to be effective if the primary source of inoculum is coming from the perennial *Urochloa* pastures growing near the wheat field. It is becoming clear that we need to know much more about the biology of *Pygt* on its non‐wheat hosts.
